# The Microenvironment-Specific Transformation of Adult Stem Cells Models Malignant Triton Tumors

**DOI:** 10.1371/journal.pone.0082173

**Published:** 2013-12-09

**Authors:** Mitra Lavasani, Jonathan B. Pollett, Arvydas Usas, Seth D. Thompson, Aaron F. Pollett, Johnny Huard

**Affiliations:** 1 Stem Cell Research Center, Children's Hospital of Pittsburgh, Pittsburgh, Pennsylvania, United States of America; 2 Department of Bioengineering, University of Pittsburgh, Pittsburgh, Pennsylvania, United States of America; 3 Department of Orthopaedic Surgery, University of Pittsburgh, Pittsburgh, Pennsylvania, United States of America; 4 Allegheny-Singer Research Institute, Pittsburgh, Pennsylvania, United States of America; 5 Pathology and Laboratory Medicine, Mount Sinai Hospital, Toronto, Ontario, Canada; 6 Department of Laboratory Medicine and Pathobiology, University of Toronto, Toronto, Ontario, Canada; 7 Department of Molecular Genetics and Biochemistry, University of Pittsburgh, Pittsburgh, Pennsylvania, United States of America; University of Minnesota Medical School, United States of America

## Abstract

Here, we demonstrated the differentiation potential of murine muscle-derived stem/progenitor cells (MDSPCs) toward myogenic, neuronal, and glial lineages. MDSPCs, following transplantation into a critical-sized sciatic nerve defect in mice, showed full regeneration with complete functional recovery of the injured peripheral nerve at 6 weeks post-implantation. However, several weeks after regeneration of the sciatic nerve, neoplastic growths were observed. The resulting tumors were malignant peripheral nerve sheath tumors (MPNSTs) with rhabdomyoblastic differentiation, expressing myogenic, neurogenic, and glial markers, common markers of human malignant triton tumors (MTTs). No signs of tumorigenesis were observed 17 weeks post-implantation of MDSPCs into the gastrocnemius muscles of dystrophic*/mdx* mice, or 1 year following subcutaneous or intravenous injection. While MDSPCs were not oncogenic in nature, the neoplasias were composed almost entirely of donor cells. Furthermore, cells isolated from the tumors were serially transplantable, generating tumors when reimplanted into mice. However, this transformation could be abrogated by differentiation of the cells toward the neurogenic lineage prior to implantation. These results establish that MDSPCs participated in the regeneration of the injured peripheral nerve but transformed in a microenvironment- and time-dependent manner, when they likely received concomitant neurogenic and myogenic differentiation signals. This microenvironment-specific transformation provides a useful mouse model for human MTTs and potentially some insight into the origins of this disease.

## Introduction

Stem cells are engaged in constant cross-talk and are influenced by the signals that they receive from their environment [1]. Cell-to-cell interaction, cell-to-tissue matrix contact, and the presence of certain factors and signaling molecules within the stem cell microenvironment regulate stem cell homeostasis and determine stem cell fate [1]–[3]. Hence, it is believed that key fate-determining events are generated by interactions between the stem cells and their local environment and are regulated in vivo by environmental factors encountered in the stem cell niche [4]. It has been suggested that the environment is a more significant factor in neural stem cell fate than the intrinsic properties of the cell [5].

Skeletal muscle has shown to contain progenitor cells that can undergo neuronal or glial lineage differentiation in vitro [6]–[9] and in vivo [10], [11]. Muscle-derived stem/progenitor cells (MDSPCs), isolated using a preplate technique in our lab, have been shown to regenerate dystrophin-positive myofibers and myocytes in a dystrophin-deficient *mdx* mouse model, participate in bone and cartilage repair after injury, and replenish the bone marrow of lethally-irradiated mice with no deleterious effects [12]–[17]. Although the true origin of MDSPCs is still unclear, recent studies suggest that they may take their origin from blood vessel walls, similar to pericytes and endothelial cells [18], [19].

Here we examine the expression of neuronal and glial cell markers by MDSPCs isolated from murine skeletal muscle under controlled culture conditions and investigate their regenerative potential after peripheral nerve injury. In addition to their ability to undergo myogenesis, MDSPCs are able to generate neurospheres and further differentiate into neuronal and glial lineages, including Schwann cells. Mice transplanted with MDSPCs immediately following a critical-sciatic nerve defect exhibited complete functional recovery, however, several weeks after regenerating the sciatic nerve, large neoplastic growths were observed. The resulting tumors were classified as malignant triton tumors (MTTs) [20]–[22] expressing myogenic, neurogenic, and glial markers.

Previously, we have reported that specific postnatal stem cells isolated from the skeletal muscle of mice, were also able to undergo malignant transformation when exposed to conflicting differentiation signals [23]. Furthermore, we found that transformation appears to be dependent on altering the balance of intrinsic and extrinsic signaling pathways and can be abrogated when the ability of a cell to undergo differentiation is removed [23]; hence, it appears that the transformation of our stem cells was differentiation-dependent. On the basis of our observations in this study, we hypothesize that MDSPCs were transformed when their intrinsic and extrinsic signaling pathways became conflicted due to multiple differentiation signals received at the wound site and that differentiating the cells prior to implantation stopped transformation. Herein, we provide a novel animal model of differentiation-dependent transformation that mimics human MTTs. We believe that this differentiation-induced transformation model is useful for studying the initiating events leading to these tumors and will lead to a better understanding of the mechanisms underlying the environmental signals and their link to stem cell transformation.

## Materials and Methods

### Ethics Statement

All animal experiments were performed with the approval of the University of Pittsburgh Institutional Animal Care and Use Committee (Animal Welfare Assurance Number A3187-01) under approved protocol #13-03.

### Cell Isolation

A modified preplate technique was used to isolate MDSPCs from skeletal muscle biopsies obtained from normal C57BL/10J mice as previously described [13], [24], [25]. MDSPCs were cultured in proliferation medium containing DMEM supplemented with 10% fetal bovine serum (FBS), 10% horse serum (HS), 1% penicillin/streptomycin (all from Invitrogen), and 0.5% chick embryo extract (CEE; Accurate Chemical). The MDSPCs were then transduced with a retroviral vector encoding a *LacZ* reporter gene containing a nuclear localization sequence (*nLacZ*) to enable donor cell tracking [26].

### Flow Cytometry

Flow cytometry (FACSAria cytometer using FACSDiva software; Becton Dickinson) was used to analyze the expression of CD34, stem cell antigen-1 (Sca-1), and CD45. Cultured MDSPCs were washed with phosphate buffer saline (PBS; Sigma-Aldrich) containing 0.5% bovine serum albumin (BSA; Sigma-Aldrich) and 0.1% sodium azide (Sigma-Aldrich). The cells were pelleted, resuspended in a 1:10 dilution of mouse serum (Sigma-Aldrich) in PBS that was supplemented with 1 µL of Fc block (rat anti-mouse CD16/CD32), and incubated for 10 min. Cells were labeled with biotinylated rat anti-CD34, FITC-conjugated anti-CD45, and a PE-conjugated rat anti-Sca-1 monoclonal antibody for 30 min. A separate fraction of cells was treated with equivalent amounts of isotype control antibodies. All fractions, including the controls, were washed and labeled with streptavidin-APC for CD34 labeling for 20 min. Just before analysis, 7-amino-actinomycin D (7-AAD) was added to each tube to exclude nonviable cells from the analysis. All antibodies, as well as streptavidin-APC and 7-AAD, were purchased from BD Biosciences. Live cell events were collected and analyzed using flow cytometry with a cell sorter (FACStar Plus or FACSAria; Becton Dickinson).

### Myogenic Differentiation

To induce myogenic differentiation, MDSPCs were plated on collagen type I-coated (Sigma-Aldrich) 24-well plates in proliferation medium at a density of 1000 cells/cm^2^ and then shifted to differentiation medium (DMEM supplemented with 2% FBS and 1% penicillin-streptomycin) 3 days after seeding. In vitro myogenic differentiation was evaluated after 5 days by immunocytochemical staining for fast myosin heavy chain (f-MyHC), a marker of late myogenic differentiation, using the protocol described below.

### Neurosphere formation and differentiation

To form neurospheres, MDSPCs were plated as a suspension on non-treated 12-well plates at a concentration of 4×10^4^ cells/mL in a medium consisting of Neurobasal™ A (Invitrogen), 100 U/mL penicillin/streptomycin, L-glutamine (2 mM; Invitrogen), supplemented with 1X B27 (Invitrogen), basic fibroblast growth factor (bFGF, 40 ng/mL; Invitrogen), and epidermal growth factor (EGF, 20 ng/mL; Sigma-Aldrich) using a similar protocol to that used by Romero-Ramos et al. [6]. Cells were maintained in this medium for 7 to 14 days, and the growth factors replaced every 3 to 4 days. The MDSPC-derived neurospheres were passaged before their diameters exceeded 100 µm, by enzymatic (using TrypLE [Gibco™]) and mechanical (with P200 plastic micropipettor) dissociation, then were reseeded in Neurobasal™ A supplemented with B27, bFGF (20 ng/mL), and EGF (10 ng/mL). To induce differentiation, the MDSPC-derived neurospheres were collected, centrifuged, and gently triturated in a "differentiation medium" containing Neurobasal™ A supplemented with 10% FBS (Invitrogen) and 1X B27 (Invitrogen). Single cell suspensions were plated on poly-D-lysine (Sigma-Aldrich, MW 70,000-150,000) coated 8-well CultureSlides (BD Falcon™) at an initial density of 5×10^4^ cells/0.7 cm^2^. Plates were checked daily to determine their state of differentiation, and 50% of the medium was replaced with fresh differentiation medium when necessary (when the medium became yellow from turning acidic). Cells were incubated at 37°C in 5% CO_2_ in a fully humidified atmosphere for 7 to 14 days.

### Immunocytochemistry

The MDSPCs, MDSPC-derived neurospheres, and tumor-derived cells (TDCs) were screened for several lineage-specific markers. Briefly, cells were fixed for 30 min in 4% paraformaldehyde (Fisher Scientific) in PBS (pH 7.4; Invitrogen), washed, and incubated for 10 min at room temperature with PBS plus 0.3% Triton X-100 to permeabilize the cell membrane. They were blocked with 10% normal donkey serum (DS; Jackson ImmunoResearch Laboratories) or goat serum (GS; Vector) for 60 min. Cells were incubated at 4°C overnight with the following primary antibodies (in 2.5% DS or GS): nestin (1∶1000, goat; Santa Cruz Biotechnology Inc.), neuronal nuclei (Neu-N, 1∶100, mouse; Chemicon), β-tubulin III (TU-20, 1∶500, mouse; Chemicon), neurofilament (NF, 150 kD, 1∶500, rabbit; Chemicon), S100 (1∶500, rabbit; Sigma-Aldrich), 2',3'-cyclic-nucleotide 3'-phosphodiesterase (CNPase, 1∶200, mouse; Sigma-Aldrich), glial fibrillary acidic protein (GFAP, 1∶500, rabbit; Chemicon), oligodendrocyte specific protein (OSP, 1∶200, rabbit; Abcam), myelin basic protein (MBP, 1∶500, mouse; Chemicon), Oligodendrocyte marker O4 (1∶200, mouse; Chemicon), NG2 Chondroitin Sulfate Proteoglycan (1∶200, rabbit; Chemicon), NGFR p75 (c-20, 1∶200, goat; Santa Cruz Biotechnology Inc.), or TrKA (763, 1∶500, rabbit; Santa Cruz Biotechnology Inc.) The cells were then exposed to the appropriate secondary antibodies, donkey anti-mouse or rabbit IgG-AlexaFluor® 594- or 488- conjugated (1∶500; Molecular Probes) for 30 min, and rabbit anti-goat IgG, Cy3-conjugated (1∶1000; Sigma-Aldrich) for 60 min. For desmin and f-MyHC immunocytochemical staining, the cells were fixed and blocked with 10% HS (Vector) and incubated with the primary antibody (both at 1:200, mouse; Sigma-Aldrich) in 2.5% HS at 4°C overnight. After gentle washing, the cultures were incubated at room temperature for 45 min with the secondary antibody, biotinylated anti-mouse IgG (1∶250; Vector), and subsequently incubated with streptavidin-conjugated Cy3 (1∶500; Sigma-Aldrich) at room temperature for 20 min. To visualize the nuclei, all cultures were incubated with 4′, 6′ diamidino-2-phyenylindole (DAPI, 100 ng/mL; Sigma-Aldrich) for 10 minutes. Culture slides (BD Falcon™) were mounted with Vectashield® medium (Vector). Brightfield, phase contrast, and fluorescent images were taken using a Leica DMIRB microscope equipped with a Retiga 1300 digital camera (Q imaging) and Northern Eclipse software system (v. 6.0; Empix Imaging, Inc.). Cells were systematically quantified in at least 15 fields across the slides or culture dish wells from four to six independent experiments. Controls included omitting the primary antibodies during the procedure.

### Cell Transplantation

To examine myogenic differentiation in vivo, a viable single cell suspension of 2×10^5^ MDSPCs or MDSPC-derived neurospheres (14 days in culture) were suspended in 20 µL of PBS and injected into the gastrocnemius muscle of 6- to 8-week-old *mdx* mice (C57BL/10ScSn DMD*mdx*/J; Jackson Laboratories) via an Ultra-Fine II syringe (BD BioSciences). At 2 and 17 weeks post-transplantation, mice were sacrificed and the gastrocnemius muscles were harvested and flash frozen in liquid nitrogen-cooled 2-methybutane. Serial cryosections, 10 µm in thickness, were prepared from frozen muscles for immunohistochemical analysis. Muscle regeneration was quantified by manually counting the number of dystrophin-positive myofibers in a section containing the largest graft and calculating the regeneration index.

Neurogenic differentiation in vivo was examined by creating a 7 mm sciatic nerve defect in 6- to 8-week-old severe combined immune deficiency (SCID) mice. Immediately thereafter, a viable single cell suspension of 3×10^5^ MDSPCs, MDSPC-derived neurospheres, or an equal volume of PBS (15 µL), was injected onto the proximal and distal nerve stumps. Mice from each group were sacrificed as early as 6 weeks after transplantation or maintained for later time points (up to 15 weeks) to check for possible tumor formation. The hind limbs, including the sciatic nerve, were harvested, frozen, and cryosectioned for further analysis.

### Soft Agar Analysis

Cells were suspended in DMEM over a 0.6% agar (Sigma) underlay at concentrations of 100, 500, and 1000 cells/well on a 12 well plate in triplicate. The cultures were allowed to grow for 2 weeks and then colonies were scored as a % (number of colonies/100 cells).

### Cell Cycle Analysis

Cells were trypsinized and washed in PBS prior to fixation in ethanol (final concentration –70%) for 15 minutes on ice. Following fixation, the cells were pelleted and resuspended in a solution containing 50 µg/mL Propidium iodide (PI), 0.1 mg/mL RNase A and 0.05% Triton X-100. The cells were incubated for 40 min at 37°C and then centrifuged and resuspended in 500μL PBS and analyzed by flow cytometry.

### Cytogenetic Analysis

Cytogenetic analysis was carried out by the University of Pittsburgh Cancer Institute Cytogenetics Facility. The cells from both the MDSPC (passage 36) and TDC (passage 12) populations were harvested following mitotic arrest with a 2 h treatment with Colcemid^TM^ (0.1 µg/ml, Irvine Scientific). The cells were then incubated in hypotonic KCl (0.075M) for 30 min at 37°C and fixed in 3:1 methanol: glacial acetic acid for 20 minutes, followed by two washes in fixative. Slides were prepared from the cell suspensions and incubated overnight at 60°C. Chromosomes were trypsin-Giemsa banded and 20 cells were counted and analyzed from each cell culture.

### Real-Time PCR

To quantify the NF1 gene, we used real-time PCR to determine the threshold cycle (Ct) number and normalized to Glyceraldehyde 3-phosphate dehydrogenase (GAPDH) as an endogenous DNA control. *Nf1* mRNA was detected by RT-PCR using forward primer 5′-GTATTGAATTGAAGCACCTTTGTTTGG-3′ and reverse primer 5′-CTGCCCAAGGCTCCCCCAG-3′, 35 cycles at the melting temperature of 60°C. While *GAPDH* levels were determined using forward primer 5′- GTCGTGGAGTCTACTGGTGTC -3′ and reverse primer 5′- GAGCCCTTCCACAATGCCAAA -3′, 35 cycles at the melting temperature of 60°C. All PCR reactions were performed on a LightCycler 480 II (Roche Applied Science, Basel, Switzerland). Experiments were performed with triplicates for each data point. Total RNA was isolated from our cells using Qiagen's RNeasy Mini Kit as per manufacturer’s instructions. For real-time PCR, 1 µg of total RNA was reverse transcribed to cDNA using Superscript III following the manufacturer's protocol (Invitrogen). Real-time qPCR was performed on 2 ng of cDNA using SYBR Green (Roche) and a Light Cycler 480II (Roche) in accordance with the manufacturer's instructions. All values were normalized to *GAPDH* expression.

### DNA sequencing

Exons 4 to 9 of the *Trp53* gene were amplified by PCR. The PCR products were run out on 1.5% agarose gels. Amplifications were directly cut from the gel and extracted using Qiagen Gel Extraction kit as per manufacture’s protocol. Gel-purified PCR products were sequenced using the BigDye Terminator Cycle Sequencing Ready Reaction version 1.1 (Applied Biosystems, Foster City, CA) and run on an ABI Prism 3730 sequencer (Perkin Elmer, Wellesley, MA). Data were analyzed using Sequencher software version 4.1.4 (Gene Codes, Ann Arbor, MI) by comparison to a reference sequence (GenBank, NM_000546). Primer sequences and PCR amplification conditions are available on request.

### Histochemistry

Sections were fixed in 1% glutaraldehyde (Sigma-Aldrich), and stained for *nLacZ* expression (using X-Gal solution 3 hours at 37°C) for donor cell tracking and then counterstained with eosin [27]. Masson’s trichrome staining was performed per the manufacturer’s protocol (Masson’s trichrome stain kit, K7228; IMEB Inc.) to determine the amount of collagen content in the fibrotic tissue. Muscle fibers are stained red, collagen is stained blue, and nuclei are black.

### Immunohistochemistry

Cryosections were immunostained for FluoroMyelin (Molecular Probes) according to the protocol provided by the manufacturer, and then fixed in 4% PFA, blocked with 5% DS for 1 h, and incubated with a primary antibody against NF (1∶300, rabbit; Chemicon), for 2 h at room temperature. We then labeled the cryosections with anti-rabbit secondary antibody conjugated with AlexaFluor® 594- or 488 for 20 min.

To colocalize donor cells with the glial cell marker CNPase, tissues were fixed with 4% formalin and blocked with horse serum for 1 h at room temperature. Sections were then incubated overnight at 4°C with anti-β-galactosidase (1∶200, mouse; Abcam) primary antibody. The next day, the sections were incubated with biotinylated horse anti-mouse IgG (1∶250; Vector) for 1 h, followed by a 20 min incubation with Streptavidin-conjugated Cy3 (1∶500; Molecular Probes) to detect the β-galactosidase. Tissues were blocked again with horse serum for 30 min, incubated with anti-CNPase (1:200, mouse; Sigma-Aldrich) primary antibody for 2 h at room temperature, and then with biotinylated horse anti-mouse IgG (1∶250; Vector) for 1 h, followed by a 20 min incubation with Streptavidin-conjugated FITC (1∶500; Molecular Probes) for CNPase detection. All sections were stained with DAPI (100 ng/mL; Sigma-Aldrich) for 10 min at room temperature to identify cell nuclei. For negative controls, the primary antibody was omitted.

To colocalize donor cells with glial cell marker, GFAP, tissues were fixed with 4% formalin and blocked with horse serum for 1 h at room temperature. Sections were then incubated overnight at 4°C with anti-β-galactosidase (1∶200, mouse; Abcam) and anti-GFAP (1∶500, rabbit; Chemicon) primary antibodies, followed by biotinylated horse anti-mouse IgG (1∶250; Vector) for 1 h, and a 20 min incubation with Streptavidin-conjugated Cy3 (1∶500; Molecular Probes) to detect the β-galactosidase. An incubation of 20 min with AlexaFluor® 488-conjugated anti-rabbit (1∶500; Molecular Probes) secondary antibody was used to detect GFAP immunoreactivity.

To colocalize donor cells with Sca-1, sections were fixed with 4% PFA for 7 min and stained using M.O.M. kit. Briefly, tissues were blocked with M.O.M.™ Mouse Ig Blocking Reagent for 1 hr at room temperature. To block non-specific binding, Avidin/Biotin block kit (Vector) was used following the manufacturer’s instructions. After 5 minutes incubation with M.O.M.™ diluent, sections were incubated with β-galactosidase (1:200, mouse; Abcam) and Sca-1 (1∶200, rat; BD) for 90 min at room temperature. The sections were then rinsed in PBS and incubated with biotinylated horse anti-mouse IgG (1∶200; Vector) in diluent for 15 min. This was followed by a 20 min incubation with streptavidin-conjugated 594 (1∶500; Sigma-Aldrich) for β-galactosidase. The sections were then rinsed and incubated with biotinylated goat anti-rat IgG (1∶250; Vector) in diluent for 20 min and followed by a 20 min incubation with streptavidin-conjugated 488 (1∶500; Sigma-Aldrich) for Sca-1. DAPI was used to identify cell nuclei. For negative controls, the primary antibody was omitted.

Tumor sections were stained with Vectastain® *Elite* ABC kit (Vector Laboratories) following the manufacturer's protocol with anti-mouse or -rabbit IgG appropriate to primary antibodies against NF (1∶500, rabbit; Chemicon), S100 (1∶500, rabbit; Sigma-Aldrich), and desmin (1:200, mouse; Sigma-Aldrich). Slides were then developed using the peroxidase 3, 3′-diaminobenzidine (DAB) substrate kit (Vector Laboratories). For the purpose of double labeling, a sequential staining of each primary antibody was performed using anti-rabbit IgG Vectastain ABC kit (Vector Laboratories). The slides were developed using DAB Substrate Kit (brown) for NF and VIP Substrate Kit (purple) for S100 (both from Vector Laboratories).

For dystrophin staining, the gastrocnemius muscles injected with MDSPCs or TDCs were harvested 14 days after transplantation, flash frozen in liquid-nitrogen-cooled 2-methylbutane, and serially cryosectioned (10 µm). Sections were fixed with 5% Formalin for 5 min, rinsed with PBS, blocked with 10% DS for 60 min, and then incubated with anti-dystrophin (1∶300, rabbit; Abcam) primary antibody in 2.5% DS for 60 min. Next, the sections were washed, then incubated with secondary antibody, AlexaFluor® 594-conjugated anti-rabbit (1∶500; Molecular Probes), for 20 min. To visualize the nuclei, sections were incubated with DAPI for 10 minutes. A Nikon Eclipse E800 microscope, equipped with a Spot digital camera and software system (v. 3.0.4; Diagnostic Instruments), was used for capturing images to quantify the regenerative index (the number of dystrophin-positive fibers in the host muscle per 10^5^ transplanted donor cells).

### Functional Assessment

Functional recovery of the sciatic nerve in mice was evaluated by measuring walking tracks, using a 6×46 cm corridor (straight maze) lined with white paper (Benchkote; Cardinal) and open at one end to a darkened compartment. The hind paws of the animals were pressed into the surface of a black waterproof inkpad. The animals were then walked multiple times to obtain measurable footprints over a 14 week time period. The animals’ feet were immediately washed in lukewarm water at the end of the tests. We scanned the collected paw prints with a Microtek 9800XL scanner and evaluated the tracks for toe spread and print length using the Northern Eclipse software system. We derived factors for each parameter to calculate the sciatic functional index using a protocol previously described and validated by Inserra *et al.* for mice [28].

### Statistical Analysis

The Student’s *t*-test or the Mann-Whitney Rank Sum test (where appropriate) were used for direct comparisons between treatment and control groups using SigmaStat (Jandel Scientific v2.0). A *P* value of <0.05 was regarded as significant.

## Results

### MDSPC isolation and myogenic differentiation

Muscle-derived stem/progenitor cells (MDSPCs) were isolated from skeletal muscles of female newborn C57BL/10J mice using a modified preplate technique [13], [24], [25]. Similar to previously reported isolates [13], most of these cells were positive for the stem cell surface markers CD34 (88.5%) and Sca-1 (87%), showing 78% CD34/Sca-1 double-positive cells by flow cytometry. Very few cells expressed the myogenic cell marker desmin (<1.5%) following immunofluorescence staining. The cells were initially plated at a density of 250 cells/cm^2^ at 20-30% confluency and transduced with a retroviral vector containing an *nLacZ* reporter gene [12], [13], [16] for donor cell tracking. The cells were subcloned to obtain a population of MDSPCs that was over 90% β-gal-positive (data not shown). We found no significant difference in cell marker expression compared to the parental MDSPCs suggesting the stability of these cells in vitro.

**Figure 1:**
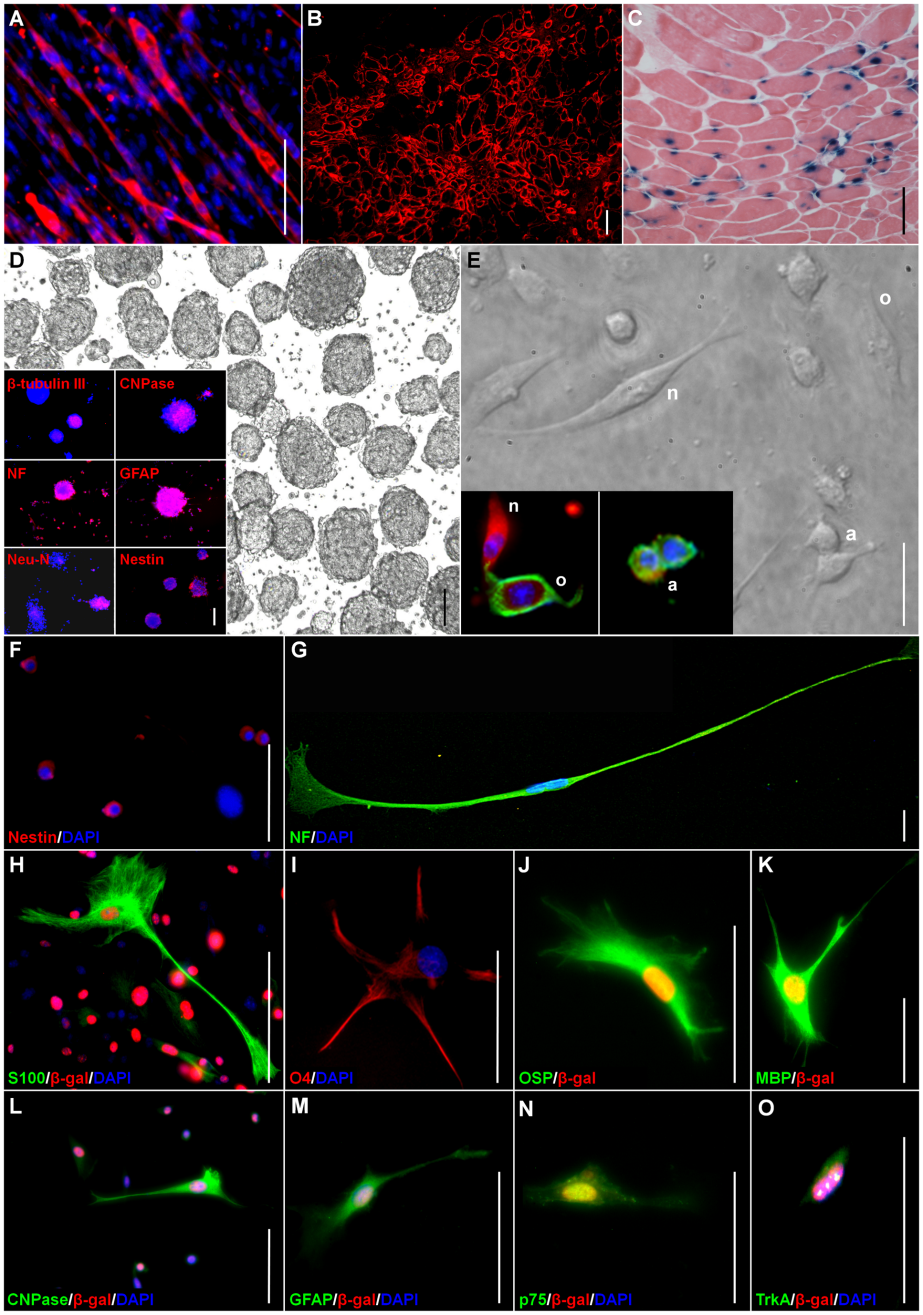
MDSPCs exhibit myogenic, neurogenic, and glial differentiation. (**A**) Ten days after culture in low serum differentiation medium, MDSPCs differentiated into multinucleated myotubes expressing f-MyHC (red) (overlaid on nuclear counterstain [DAPI; blue]). (**B**) Many regenerated dystrophin-positive myofibers (red) are observed in gastrocnemius muscle cryosections of *mdx* mice (*n*  =  10) 14 days after injection. (**C**) The *nLacZ*-positive donor-derived MDSPCs (blue) could be detected in regenerated myofibers 17 weeks after injections. (**D**) MDSPCs were able to generate neurospheres (three-dimensional non-adherent clusters of cells) in enriched-culture medium within 7 to 10 days in culture (brightfield image). MDSPC-derived neurospheres express the neuronal cell markers β-tubulin III, NF, Neu-N, the myelin producing oligodendrocytes marker CNPase, the astrocytic marker GFAP, and retained their nestin (a neuroepithelial progenitor marker) expression (inset). Antibodies used are visualized in red with the nuclear stain, DAPI seen in blue. (**E**) The MDSPC-derived neurospheres can be further differentiated into neurons (β-tubulin [red], n), oligodendrocytes (NG2 [green], o), and astrocytes (GFAP [green], a) (inset). MDSPC-derived neurospheres expressed (**F**) nestin and were differentiated further into cells that expressed markers found on neuronal and glial (including oligodendrocytes, astrocytes, and Schwann cells) cells, including (**G**) NF, (**H**) S100, (**I**) O4, (**J**) OSP, (**K**) MBP, (**L**) CNPase, (**M**) GFAP, (**N**) the neurotrophin receptor p75, and (**O**) the tyrosine kinase receptor TrkA (all green, except O4 [red]). The nuclear stain DAPI seen in blue and β-gal-positive nuclei in red (for H and J-O). Data represent four to six independent experiments. Scale bars represent 100 µm (**A-D**) or 50 µm (**E-O**).

As expected, MDSPCs fused and formed multinucleated myotubes expressing f-MyHC (Fig. 1A). In order to further explore the myogenic potential of MDSPCs, their ability to regenerate dystrophin-positive muscle fibers after intramuscular implantation in dystrophin-deficient *mdx* mice was examined (*n* = 10). Fourteen days post-implantation, many dystrophin-positive myofibers could be found within the injected muscle (Fig. 1B). Furthermore, donor *nLacZ*-positive nuclei could be observed within the regenerated myofibers 17 weeks (120 days) post-implantation (Fig. 1C).

### MDSPCs can undergo neuronal and glial differentiation in vitro

The MDSPCs were screened by immunocytochemistry for a series of neurogenic and glial cell markers prior to differentiation. Undifferentiated MDSPCs showed phenotypic heterogeneity by their expression of cell markers that label neuroepithelial progenitors (nestin, 91±4.9%), oligodendrocytes and Schwann cells (CNPase, 88±9.0%), and postmitotic neurons (NF, 53±14.0%); however, they were negative for other cell markers of neuronal maturity, such as neuronal nuclei, Neu-N. When MDSPCs were cultured in defined serum-free medium supplemented with epidermal growth factor (EGF) and basic fibroblast growth factor (bFGF) [6], they generated free-floating clusters of cells (MDSPC-derived neurospheres) within 7 to 10 days (brightfield; Fig. 1D). Many of the cells on the outer surface displayed microspikes—a characteristic of neurospheres evident at higher magnification [6] (data not shown). These MDSPC-derived neurospheres expressed neuronal markers (β-tubulin III, NF, and Neu-N), a marker for myelin producing oligodendrocytes and Schwann cells (CNPase), and the astrocytic marker GFAP (inset; Fig. 1D). Though these MDSPC-derived neurospheres expressed neuronal and glial lineage commitment markers, they also retained expression of the neuroendothelial progenitor marker nestin (inset; Fig. 1D).

The MDSPCs, unlike some neural stem cells derived from the developing brain [29], appear to require exogenous factors to trigger commitment to a neurogenic cell lineage. A negatively charged culture surface, high serum levels, and eliminating mitogens, were all required to further induce neuronal and glial differentiation of the MDSPC-derived neurospheres. After 3 days in culture, the neurospheres differentiated into a variety of cell types with diverse morphologies; including small and round, large and flat, spindle-shaped, and round cell bodies with thin extended processes (phase contrast; Fig. 1E). Over 70% of cells were positive for the neuronal markers β-tubulin (n; Fig. 1E) and NF (data not shown), 88% were positive for the astrocyte marker GFAP (a; Fig. 1E), 22% expressed the immature oligodendrocyte marker NG2 (o; Fig. 1E), and 65% displayed the mature oligodendrocyte marker O4 (data not shown). The simultaneous expression of both neuronal and astrocyte cell markers has been observed previously in vitro in hippocampal stem cells treated with bFGF in serum-free medium [30], in the rat embryonic striatum at early stages of differentiation [31], and in immortalized embryonic mesencephalic mouse cells [32]. Similarly, postnatal forebrain neural progenitors can display mixed glial and neuronal properties in vitro when they co-express neuron-specific enolase (NSE), NF, and GFAP [33]. Thus, co-expression of glial and neuronal cell markers seems to reflect multipotentiality at early stages of neurogenic differentiation in vitro. MDSPC-derived neurospheres expressed nestin (Fig. 1F) and could be further differentiated into neuronal, glial, and Schwann cell lineages positive for NF (Fig. 1G), S100 (Fig. 1H), O4 (Fig. 1I), OSP (Fig. 1J), MBP (Fig. 1K), CNPase (Fig. 1L), GFAP (Fig. 1M), p75 (Fig. 1N) and TrkA (Fig. 1O). Taken together these results indicate that MDSPC-derived neurospheres can differentiate into neurogenic and glial lineages, including Schwann cells, in vitro.

### MDSPCs differentiate into Schwann cells in vivo and participate in the regeneration of the injured peripheral nerve

**Figure 2:**
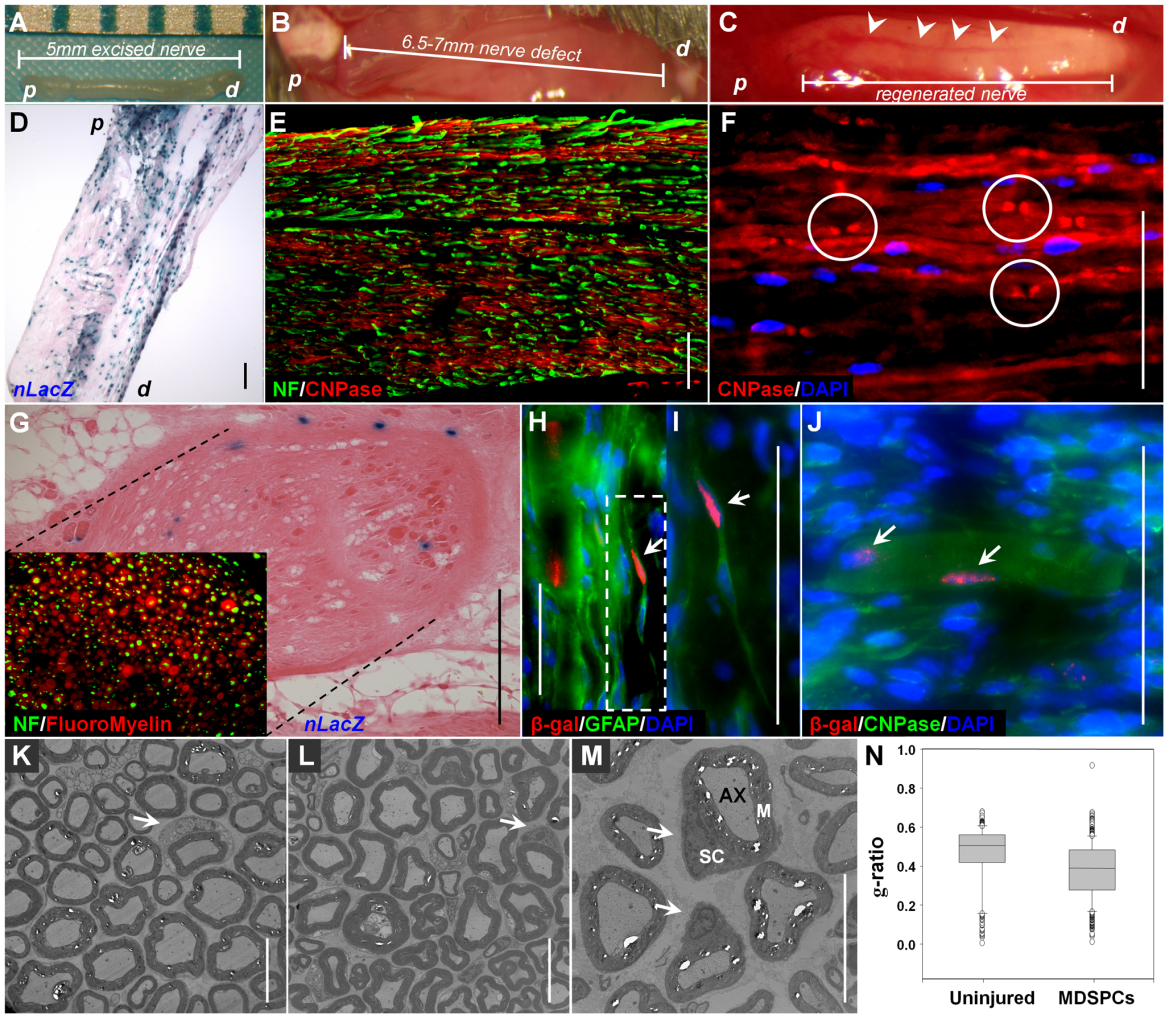
Transplanted MDSPCs foster repair of critical-size sciatic nerve defects. (**A**) A 4- to 5 mm segment of the sciatic nerve was removed from the hind limb of each mouse, (**B**) resulting in a 6.5 to 7 mm defect. (**C**) Following transplantation of MDSPCs into the defect, complete regeneration from proximal to distal end was observed *(n*  =  28). Blood vessel networks (arrowheads) were also present around all regenerated nerves. (**D**) Many *nLacZ*-positive cells (blue) were observed between weeks 5 and 9 following injury. “*p*” corresponds to the proximal stump and “*d*” to the distal stump. (**E**) The regenerated nerve exhibited both NF (green) and CNPase (red) immunoreactivity. (**F**) CNPase (red) staining of the regenerated sciatic nerve revealed nodes of Ranvier-like structures (white circles). (**G**) Cross-sections of regenerated nerve showed *nLacZ*-positive cells (blue) and exhibited NF-positive axons (green, inset) encompassed by FluoroMyelin-positive cells (red, inset). (**H, I, J**) Colocalization of β-gal (red) with (**H, I**) GFAP (green) or (**J**) CNPase (green), and DAPI (blue) suggests possible differentiation of the MDSPCs into Schwann cells (double-positive cells denoted by arrows). (**K-M**) Electron microscopy of semi-thin cross-sections of (**K**) non-operated (uninjured) control, and (**L, M**) MDSPC-regenerated peripheral nerve 10 weeks after implantation, show a high number of myelin-producing Schwann cells. Arrows indicate Schwann cells surrounding the myelinated axon. “Sc” corresponds to Schwann cells, “M” to myelin sheath, and “Ax” to axons. (**N**) Graphical quantification of the g-ratio (axonal area: myelinated fiber area) represents the median values of both uninjured and MDSPC-regenerated nerves (*P*<0.001, Mann-Whitney Rank Sum Test). Sciatic nerve regeneration studies represent three independent experiments. The morphometric parameters represent results from 5 mice (2 controls and 3 treated) and analysis of 1000 fibers. Scale bars represent 100 µm (**D, E, and G**) or 10 µm (**F, H-M**).

To test the ability of MDSPCs to participate in the regeneration of the peripheral nerve following injury, the cells were implanted into a critical-size sciatic nerve defect (a defect unable to undergo autonomous healing) in mice. The removal of a 4 to 5 mm nerve segment (Fig. 2A) resulted in a ∼6.5 to 7 mm critical-size defect due to the retraction of the nerve ends (Fig. 2B). The vehicle (PBS) group exhibited no regeneration (*n* = 24); some nerve sprouting on the proximal side of the defect and neuroma formation (a bulb-shaped thickening created by ineffective and unregulated nerve regeneration) were the main outcomes (data not shown). However, complete sciatic nerve regeneration was observed by 5 to 9 weeks in all of the MDSPC implanted mice (*n* = 28; Fig. 2C). Notably, blood vessel networks were also present around all regenerated nerves (arrowhead; Fig. 2C). The regenerated nerves contained many donor *nLacZ*-positive cells (Fig. 2D) and exhibited both NF-(green) and CNPase immunoreactivity (red; Fig. 2E). Furthermore, the regenerated nerve contained short nerve segments with node-like discontinuities in the myelin sheet (CNPase, red) that resembled nodes of Ranvier-like structures (white circles; Fig. 2F). Cross sections of regenerated nerves exhibited many regenerated NF-positive axons (green) encompassed by FluoroMyelin (red), indicating the presence of myelin-producing Schwann cells that form the myelin sheaths surrounding the regenerated axons (Fig. 2G). To confirm the differentiation of donor cells to glial lineage, we were able to detect β-gal positive cells (red nucleated cells) expressing the glial and Schwann cell markers GFAP (green; Fig. 2H, 2I) and CNPase (green; Fig. 2J) along the regenerated sciatic nerve.

Quantitative measurement of morphometric parameters, such as number of myelinated axons, myelin thickness, myelinated fiber area, axonal area, and g-ratio (axonal area : myelinated fiber area) [34] were compared between uninjured control (*n* = 2) and MDSPC-transplanted nerves (*n* = 3) in images taken with a transmission electron microscope (TEM; Fig. 2K-M). Ten weeks after implantation, the mid-section of the regenerated sciatic nerve (MDSPC-transplanted) contained an average of 576±33 myelinated axons compared to 387±12 in the uninjured nerves (*P*<0.001). Myelinated fiber area and myelin thickness were assessed as a measure of regenerated fiber maturation. Myelinated axons in the MDSPC-transplanted group showed a median cross-sectional area of 8.53 µm^2^, versus 13.8 µm^2^ for the uninjured group (*P*<0.001, graph not shown) and a median myelin thickness area of 5.07 µm^2^ as compared to 7.1 µm^2^ for the uninjured control group (*P*<0.001, graph not shown). The median and 25^th^ to 75^th^ percentiles of g-ratio in mid-sections of the regenerated nerves were 0.40% and 0.28% to 0.48%, respectively, versus the uninjured control group which was 0.50% and 0.42% to 0.56%, respectively, (*P*<0.001; Fig. 2N). The increase in the number of myelinated axons following MDSPC transplantation may be the result of axonal branching leading to an increased target organ reinnervation and thus less axonal dieback [35]. Although the regenerated peripheral nerves are significantly different from the uninjured nerves for all morphometric parameters mentioned, we believe the high number of myelin-producing Schwann cells (arrows; Fig. 2M) and the low g-ratio (Fig. 2N) indicate thick myelination of the regenerated axons, highlighting the process of myelination exhibited in the MDSPC-transplanted group.

**Figure 3:**
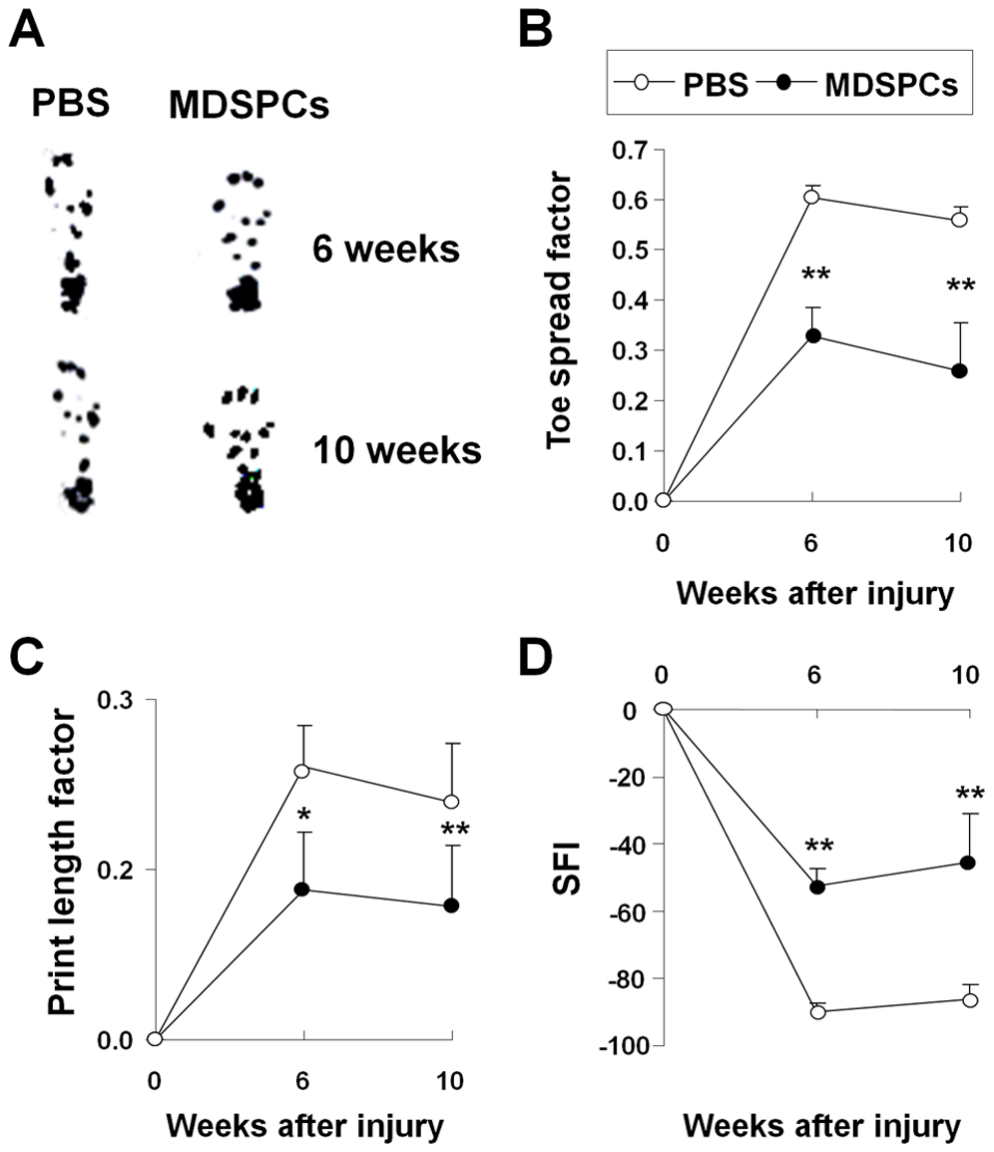
Transplanted MDSPCs assist in functional recovery of critically-sized sciatic nerve defects. (**A**) A depiction of representative paw prints from control- and MDSPC-implanted mice at 6 and 10 weeks post-implantation. (**B-D**) Quantification of paw print analyses, indicating that MDSPC transplantation increases the ability of the mice to walk normally, displayed a (**B**) decrease in toe spread factor, (**C**) decrease in print length factor, and (**D**) an increase in SFI (sciatic functional index) compared to PBS-treated mice. Thirty-five paw prints were analyzed per group for each time point. Error bars indicate s.e.m. (*P<0.05 and **P<0.001, Mann-Whitney Rank Sum Test).

Functional recovery of regenerated sciatic nerves was determined by monitoring mice on a walking track. According to paw print analysis, MDSPC-treated mice improved their walking pattern, evidenced by wider toe spread and shorter print length (Fig. 3A). Further quantifications of paw prints revealed functional recovery as determined by a decrease in toe spread factor (Fig. 3B), a decrease in print length factor (Fig. 3C), and an increase in sciatic functional index (SFI; Fig. 3D), when compared to PBS-treated control mice. Cell transplantation significantly decreased the toe spread factor (0.326±0.058 versus 0.604±2.51 for PBS-treated mice, *P*<0.01) and print length factor (0.132±0.05 versus 0.24±0.036 for PBS-treated mice, *P*<0.01) at 6 weeks post-transplantation. These differences became more evident at 10 weeks, (toe spread 0.259±0.097 versus 0.558±0.026 for PBS-treated mice, *P*<0.01 and print length 0.117±0.054 versus 0.209±0.052 for PBS-treated mice, *P*<0.01). The first signs of motor nerve recovery could be observed at 6 weeks post-transplantation for the MDSPC-transplanted groups (SFI: –52.4±5.30) versus PBS-treated mice (SFI: –90±2.51, *P*<0.001). After 10 weeks, the MDSPC-transplanted groups (–45.2±14.2, *P*<0.01) demonstrated further improvement in comparison to the PBS-treated control groups (–86.1±4.28).

### MDSPCs undergo environment-specific transformation 11 weeks post-transplantation into a sciatic nerve defect

**Figure 4:**
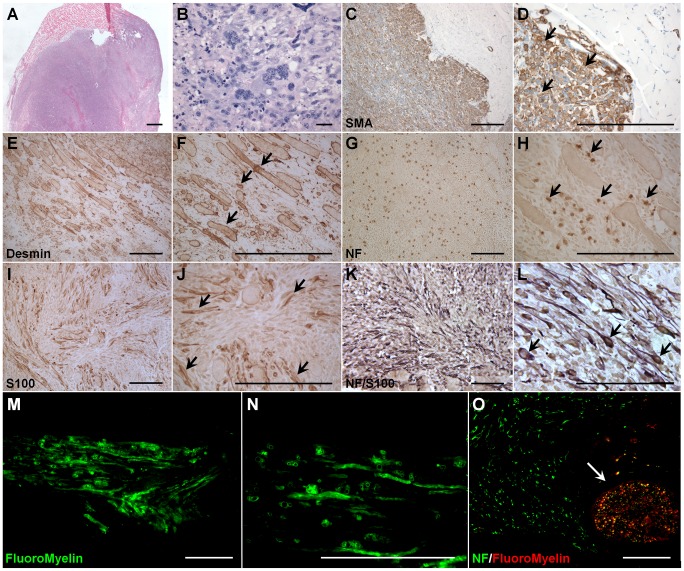
Between weeks 11 and 13, approximately 70% of the mice (*n*  =  28) implanted with MDSPCs formed large neoplastic growths. (**A, B**) Representative image of hematoxylin and eosin staining of tumors that formed in mice implanted with MDSPCs. (**C-L**) The resulting tumors were classified as malignant peripheral nerve sheath tumors with rhabdomyoblastic differentiation (Triton tumors) by showing positivity for (**C, D**) smooth muscle actin (brown), (**E, F**) desmin (brown), (**G, H**) NF (brown), and pockets of (I, J) S100 (brown), (**K, L**) as well as cells positive for both NF (brown) and S100 (purple). Positive cells (brown, **C-J**) or double-positive cells (K and L) are indicated by black arrows. Images B, D, F, H, J, and L show a higher magnification image of the image they were preceded by. (**M, N**) The neoplasias were also positive for FluoroMyelin (green), showing areas of unorganized myelin deposition. (**O**) Image depicting NF (green) and FluoroMyelin (red) double-stained sections from a regenerated nerve (white arrow) in the area of tumorigenesis. Negative control sections were similarly processed without primary antibodies. Scale bars represent 100 µm.

Approximately 70% of donor cells (n*LacZ*-positive) present in the regenerating sciatic nerve at 5 weeks post-implantation were Sca1-positive, indicating that a large proportion of donor MDSPCs remain as progenitor cells weeks after transplantation (Figure S1). Although complete nerve regeneration was observed 6 weeks post-implantation, 70% of the mice (*n* = 28) formed large neoplastic growths between weeks 11 and 13 (Fig. 4A, B). As these neoplasias were composed almost entirely of *nLacZ*-positive cells (data not shown), the neoplasias were highly invasive and positive for the myogenic markers α-smooth muscle actin (Fig. 4C, D) and desmin (Fig. 4E, F). The tumors were also positive for the neurogenic marker NF (Fig. 4G, H), contained focal areas positive for S100 (Fig. 4I, J), areas that are double-positive for both NF (brown) and S100 (purple; Fig. 4K, L), and pockets of unorganized myelin deposition, as seen by FluoroMyelin staining (Fig. 4M, N). According to histological features, the resulting tumors were classified as malignant peripheral nerve sheath tumors (MPNSTs) with rhabdomyoblastic differentiation, otherwise known as "Triton tumors" [20]–[22]. Notably, while tumorigenesis was observed, nerve regeneration was still apparent (white arrow indicating regenerated nerve; Fig. 4O), implying that tissue regeneration and cellular transformation may not be mutually exclusive events.

### Cells isolated from MDSPC-derived tumors grow as neurosphere-like structures

**Figure 5:**
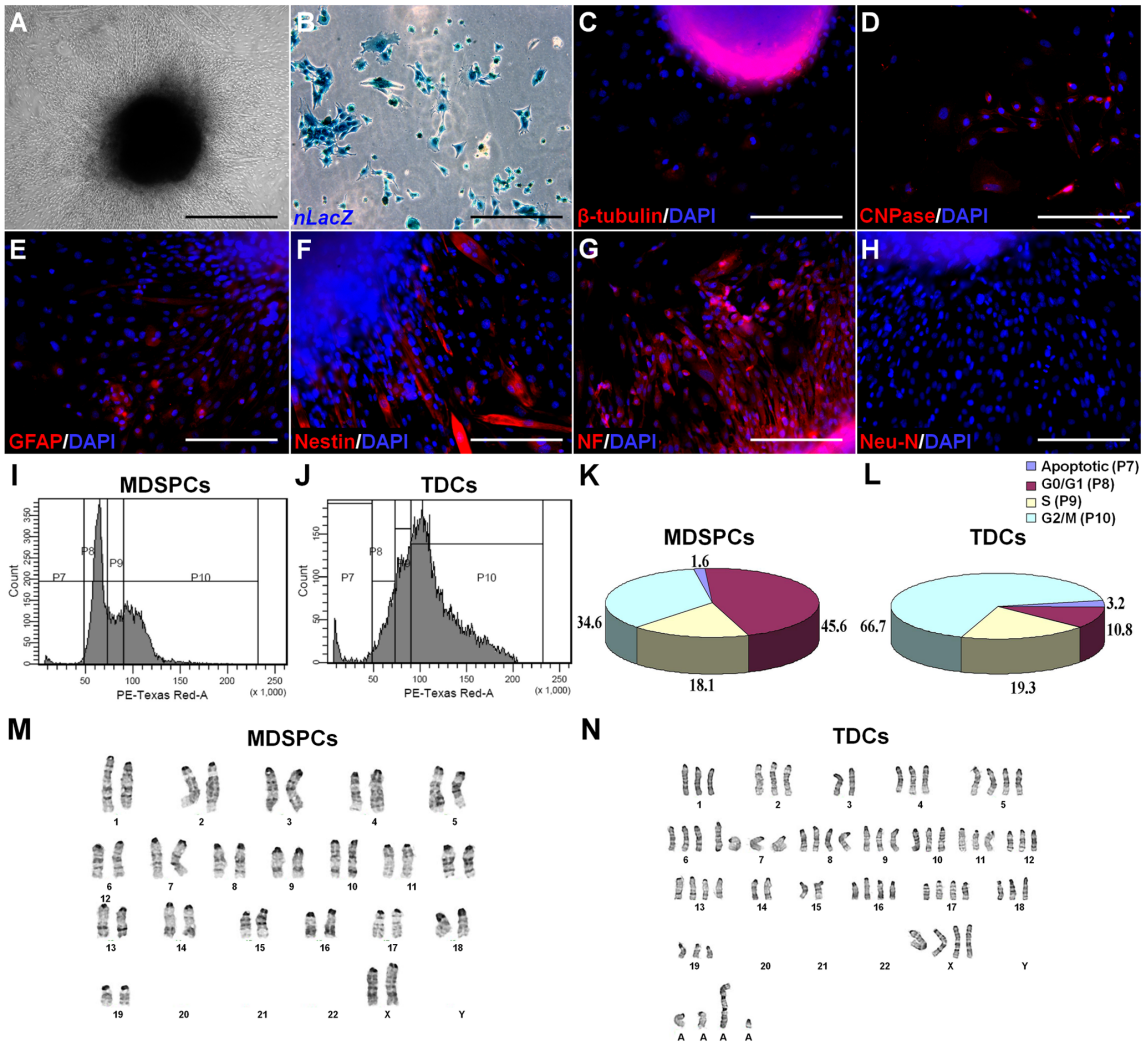
TDCs maintain their neurogenic and myogenic differentiation potential in vitro. (**A**) TDCs grew as neurosphere-like structures in the absence of neurogenic medium, and (**B**) were *nLacZ*-positive (blue). (**C-H**) Immunofluorescent analysis of these spontaneously-occurring neurosphere-like structures demonstrated that they were positive for (**C**) β-tubulin III, (**D**) CNPase, (**E**) GFAP, (**F**) nestin, and (**G**) NF, but were negative for (**H**) Neu-N. For all immunofluorescence, the antibodies used are visualized in red, with the nuclear stain DAPI seen in blue. (**I-L**) Cell cycle analysis of the parental MDSPCs and TDCs was performed by FACS. Shown is the (**I, J**) DNA content and (**K, L**) graphical representation of the results, indicating the apoptotic, G_0_/G_1_, S, and G_2_/M fractions. (**M, N**) Karyotypic analysis of MDSPCs and TDCs. (**M**) Depicted is a representative karyotype from the MDSPC population (40, XX). (**N**) Karyotype of one of the TDCs that has 68 chromosomes, is hypertriploid, and expresses several unidentifiable marker chromosomes, the largest of which appears to be dicentric. Scale bars represent 100 µm.

Tumor-derived cells (TDCs) cultured in proliferation medium, grew spontaneously as neurosphere-like structures in the absence of neurogenic stimulation (Fig. 5A) and were *nLac-Z* positive (Fig. 5B). These neurosphere-like structures were positive for β-tubulin III (Fig. 5C), CNPase (Fig. 5D), GFAP (Fig. 5E), nestin (Fig. 5F), and NF (Fig. 5G), but were negative for Neu-N (Fig. 5H). Furthermore, as determined by soft agar assays, TDCs had gained the ability to grow independently of anchorage, while their parental counterparts (MDSPCs) were incapable of forming colonies (data not shown). Cell cycle analysis of the parental MDSPCs and TDCs revealed a shift of DNA content of the TDCs from the majority of cells being in the G_0_/G_1_ phase (parental 45.6% vs. TDCs 10.8%) to the G_2_/M phase (parental 34.6% vs. TDCs 66.7%), with over 50% of the TDCs possessing more than a diploid amount of DNA (Fig. 5I-L). We carried out cytogenetic analysis on both MDSPC (Fig. 5M) and TDC populations (Fig. 5N). Analysis of the MDSPC culture revealed murine chromosomes with near-diploid and near-tetraploid chromosome constitutions; no clonal structural abnormalities were observed. However, analysis of the TDCs revealed chromosome numbers ranging from 46-118. None of the TDCs had a chromosome complement that would be considered normal diploid or tetraploid. Two of the structural abnormalities observed in the TDCs appear to be clonal, since they were each observed in more than one cell. Both of these were large, unidentifiable marker chromosomes, one of which appeared to be dicentric. Nonclonal chromosome abnormalities were seen in each of the TDCs. The karyotypic variability in these cells suggests the presence of chromosomal instability [36].

### Genetic Analysis of MDSPC-derived Tumor Cells

To determine the level of *Nf1* expression, real-time PCR analysis was performed on the mouse *Nf1* gene to look at relative expression in cells pre- and post-implantation. The *Nf1* gene was expressed in both pre- and post-transplantation cells. There is a slight decrease in the *Nf1* gene expression post-implantation (normalized Ct-values of 27.45 vs. 28.8 respectively), however, this trend was not significant (p = 0.068, *t*-test). To investigate for mutations in the *Trp53 gene*, sequencing of exons 4 to 9 was performed on DNA from cells pre- and post-implantation. The sequence of the *Trp53* gene pre- and post-implantation contained no discernible transformation mutations.

### TDCs generate tumors when implanted in vivo

**Figure 6:**
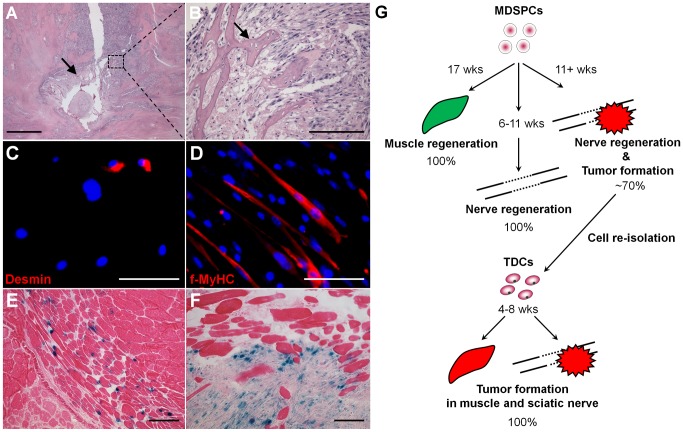
TDCs can regenerate tumors in vivo. (**A, B**) Hematoxylin and eosin staining of a tumor generated from TDCs implanted into a sciatic nerve defect in mice (*n*  =  16) demonstrates the destruction of the bone, as seen by the smooth and pink stained portion (arrows). (**C, D**) In vitro myogenic analysis demonstrates that the TDCs possess expression of (**C**) the myogenic marker desmin (red, 12%) and have the ability to form myotubes in vitro, as seen by (**D**) f-MyHC staining (red). (**E, F**) In vivo myogenic analysis demonstrates that although (**E**) parental MDSPCs (blue) showed myofiber regeneration (muscles stained with eosin) after injection into the gastrocnemius muscles of *mdx* mice and were detected up to 17 weeks without sign of tumor formation (*n*  =  10), the (**F**) TDCs (blue) implanted into the muscle formed tumor 100% of the time (*n*  =  10), and as early as 4 weeks post-implantation. (**G**) A schematic of the experimental design and results displayed above. Negative control sections were similarly processed without primary antibodies. Scale bars represent 250 µm (**A**) or 100 µm (**B-F**).

To determine whether the TDCs were able to regenerate tumors, the cells were implanted into a critical-sized sciatic nerve defect in mice (*n* = 16). Following implantation, neoplastic growths were observed between 4 and 8 weeks in all implanted mice. Furthermore, these tumors were highly aggressive and invaded all of the surrounding tissue of the leg, destroying muscle as well as bone (arrows), and were composed of poorly undifferentiated rhabdomyoblasts (small blue and pink cells) (Fig. 6A, B).

### TDCs maintained their ability to undergo myogenesis in vitro but formed tumors when implanted into the skeletal muscle

Though TDCs appear to be spontaneously neurogenic, they express the myogenic marker desmin (red, 12%; Fig. 6C) and retain their ability to form f-MyHC-positive myotubes in vitro (Fig. 6D). While the parental MDSPCs regenerated muscle fibers and showed no sign of tumorigenesis up to 17 weeks post-implantation (*n* = 10; Fig. 6E), the TDCs rapidly formed tumors when injected into the skeletal muscle of mice (*n* = 10). These tumors formed 4 weeks post-implantation in 100% of the mice and were composed of donor *nLacZ*-positive cells (Fig. 6F). A schematic representation of these results is shown in Figure 6G. Of note, injection of parental MDSPCs, subcutaneously or intravenously, exhibited no signs of tumor formation up to 1 year post-implantation (data not shown).

### In vitro differentiation of MDSPCs eliminates niche-specific transformation

**Figure 7:**
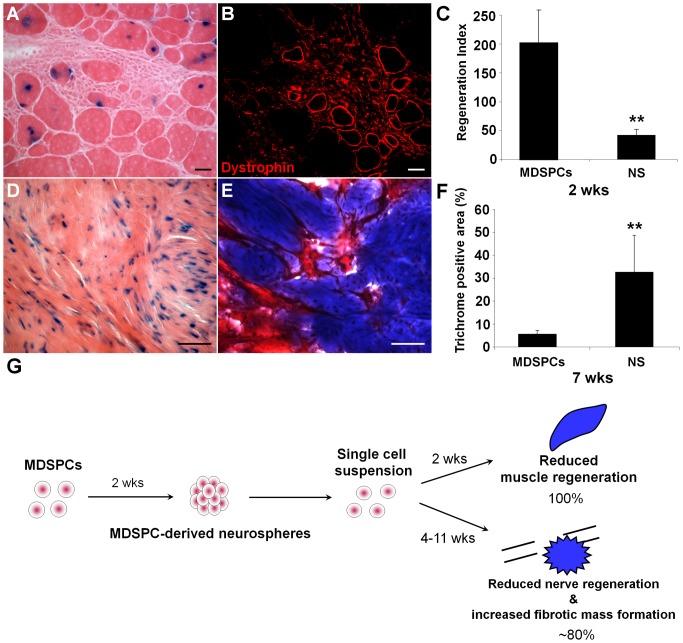
Differentiation of MDSPCs to a neurogenic lineage prior to implantation decreases their ability to respond to environmental cues and stops transformation. (**A-C**) Two weeks post-implantation MDSPC-derived neurospheres showed a reduction in muscle regeneration when compared to the parental MDSPCs. (**A**) The *nLacZ* donor-derived MDSPC-derived neurospheres (blue) could be detected in eosin stained regenerated myofibers and (**B**) showed fewer regenerated dystrophin-positive myofibers (red) compared to undifferentiated MDSPCs. (C) Graphical representation of the regeneration index (number of dystrophin positive fibers/100,000 injected cells) of parental MDSPCs and MDSPC-derived neurospheres (NS). NS-injected mice yield a lower regeneration index compared to the undifferentiated MDSPCs. Error bars indicate ± s.d. (**P<0.001, Mann-Whitney Rank Sum Test). (**D-F**) When dissociated MDSPC-derived neurospheres were implanted into a sciatic nerve defect, tumor formation was abrogated. Furthermore, 80% of mice (*n*  =  5) formed large fibrotic masses, as seen by (**D**) eosin staining with *nLacZ* donor MDSPC-derived neurospheres shown in blue, in addition to (**E**) intense collagen deposits identified with Masson’s Trichrome staining (blue). (**F**) Graphical representation of the Trichrome-positive area of parental MDSPCs and NS. Parental MDSPCs show minimal signs of fibrosis evident by a lower percentage of collagen-positive area. Error bars indicate ± s.d. (**P<0.001, Mann-Whitney Rank Sum Test). (**G**) A schematic of the experimental design and results. Scale bars represent 10 µm.

To rule out the possibility that our perceived transformation was due to the presence of pre-transformed cells within our MDSPC population, and was, in fact, due to an imbalance of intrinsic and extrinsic signaling, we altered the intrinsic predilection of the cells by differentiating them prior to implantation. If environmental cues were leading to the transformation of MDSPCs, then altering the ability of cells to differentiate to a committed lineage should abrogate transformation. Therefore, MDSPCs were cultured as neurospheres for 14 days, dissociated to a single cell suspension, and implanted into either the skeletal muscle of dystrophic mice or a critical-sized sciatic nerve defect. When the dissociated neurospheres were injected into the skeletal muscle of *mdx* mice, poor muscle engraftment was observed (Fig. 7A, B), as evident by a decrease in regeneration index (number of dystrophin positive myofibers per 100,000 injected cells) compared to parental MDSPCs (36.7±9.45 versus parental MDSPCs 180.2±49.1, *P*<0.001; Fig. 7C). This suggests that in vitro differentiation of cells towards a neurogenic lineage reduced the ability of the cells to undergo myogenic differentiation. This was expected, since we postulated that differentiation of MDSPCs prior to implantation would diminish their ability to respond to local environmental cues. More interestingly, when the dissociated MDSPC-derived neurospheres were implanted into a critical-sized nerve defect of mice, no tumor formation was observed (*n* = 5), however, 80% of the mice formed fibrotic masses positive for collagen by Masson’s trichrome staining 4 weeks post-implantation (as seen by intense collagen deposits [blue]; Fig. 7D-E), while the remaining mouse exhibited no signs of tumor formation, nerve regeneration, or fibrosis. In comparison, the implanted parental MDSPCs generated large neoplastic growths that contained almost no Trichrome-positive areas (5.63%±1.6 versus neurosphere-implanted group 32.5%±16.1, *P*<0.001; Fig. 7F). These findings are summarized in the schematic representation shown in Figure 7G.

## Discussion

In the process of investigating the potential of murine postnatal MDSPCs to regenerate peripheral nerve, we have isolated a population of stem/progenitor cells that are able to differentiate into muscle, neuronal, and glial cells depending on the environmental cues that they receive. Although these cells appear to have tremendous regenerative potential, they form neoplastic growths, classified as MTTs [22], between 11 and 13 weeks post-implantation, but only when implanted into a sciatic nerve defect. MTTs, referred to as MPNSTs with rhabdomyoblastic differentiation [20], are an uncommon malignancy in humans, which are believed to originate from the neurilemmal sheath of peripheral nerve fibers [37] or from cells associated with the nerve sheath, such as Schwann cells, perineural cells, or fibroblasts; they comprise approximately 5–10% of all soft tissue sarcomas [22], [38]. MTTs are aggressive cancers with crude two- and five-year survival rates of 33% and 12%, respectively [39], [40]. For a tumor to be classified as an MTT, it must meet the following criteria: (i) originate along a peripheral nerve, or represent a metastasis from such a tumor, (ii) have growth characteristics of a Schwann cell tumor phenotype, and, (iii) demonstrate that rhabdomyoblasts can arise from within the body of the tumor [21]. Based on the analysis of the tumors generated here and consultation with independent pathologists, the generated tumors fit the criteria to be classified as MTTs. Furthermore, MTTs have been shown to be positive for S100, desmin, muscle-specific actin, and myogenin; [21], [38] all of which are exhibited by our observed tumors. Furthermore, based on our cytogenetic analyses of primary MTTs, and as seen by others [41], it appears that the tumor-derived cells are genetically unstable, displaying more than normal modality. Similarly, the cell cycle analysis shows that the majority of these cells had increased DNA content, with 67% of the cells in the G_2_/M tetraploid state.

It was originally believed that these tumors were caused by either Schwann cells inducing muscular differentiation of other cells, or by malignant Schwann cells transforming directly into striated muscle cells [20]. This latter theory is the more widely accepted concept as it is possible that Schwann cells, being of neural crest origin, may retain a capacity for mesenchymal differentiation [40]. In 1921, Rotter postulated that adult cancers arise from the inappropriate reactivation of embryonal cell-like progenitor cells within adult tissues [42]. Herein, we posit an alternative theory and provide an experimental mouse model along with the possible explanation for the origins of Triton tumors. Based on our results, we hypothesize that Triton tumors may originate from the "inappropriate activation" of progenitor cells present in skeletal muscle that become transformed when activated and exposed to a neurogenic stimulus.

Previously, we have shown how a lack of regulation of these signals is involved in the initiation of the oncogenic potential of specific populations of MDSPCs [23]. In this model, for the spontaneous transformation of somatic stem cells, progenitor cells isolated from the skeletal muscle of mice show spontaneous microenvironment-specific transformation when expressing the osteogenic factor BMP4 [23]. The tumors generated were osteorhabdomyosarcomas and formed only when a specific population of MDSPCs was transduced with BMP4 and implanted into a myogenic environment [23]. The finding that stem cells can undergo spontaneous transformation is not unique to MDSPCs. Embryonic stem cells (ES) and testicular stem cells (TS) have been shown to form teratomas when implanted outside of their native environments [43]–[49]. Co-culturing human ESCs with immortalized human astrocytes from fetal midbrain tissue cells (making them more similar to adult progenitor cells prior to implantation) resulted in substantial and long-lasting restitution of motor function when transplanted into a Parkinsonian mouse model [50]. Interestingly, the component of the regenerated tissue that was comprised of slowly-dividing cells was potentially tumorigenic. These cells resembled a population of neuroepithelial cells that had failed to differentiate [50]. Mesenchymal stem cells (MSCs) have been shown to become tumorigenic [51]–[53] and have been implicated in childhood leukemia [54], epithelial cancers [55], and osteosarcomas [56]–[58]. It has also been shown that leukemic stem cells express antigens similar to those expressed by hematopoietic stem cells (HSCs) [59]. Furthermore, the concept of peripheral nerve regeneration inducing tumors is not new. Though peripheral nerves have the capacity to repair and restore their function, complete recovery after peripheral nerve lesion rarely occurs in the clinical setting [60], [61]. Often, as a result of improper and irregularly regenerating nerve fibers, benign (non-malignant) tumors (neuromas) develop [61].

In light of these findings, we posit that there are two distinct populations within our pool of cells− one that is actively cycling and the other that is slower to cycle (quiescent), remaining as a “reserve” pool. When these cells are implanted in a mouse sciatic nerve defect, the cycling cells undergo glial differentiation and assist in the regeneration of the peripheral nerve, while the reserve cells remain quiescent. As the cycling cells become depleted to differentiation, the quiescent cells become “activated”. However, when the healing is complete, the neurogenic/glial signals generated from the injury decrease and the newly activated reserve cells begin to respond to both the decreased neurogenic/glial signals from the injury, as well as the myogenic environmental cues (which are no longer masked by the neurogenic/glial signals). Since the reserve cells have the capacity to respond to both the environmental myogenic signals as well as the signals generated from the injury, these two signals disrupt cellular homeostasis and, in time, lead to transformation. This may explains why our model initially displays peripheral nerve regeneration, followed by transformation weeks later. This is supported by our observation that clusters of Sca1-positive progenitor donor cells are still present in the regenerating nerve before the transformation was initiated.

The previously introduced idea of a close association between wound healing and tumorigenesis by Rudolf Virchow in 1863, has been further evaluated in the past decade [62] and highlights the recent observation that adult myelinating Schwann cells in neurofibromatosis type 1 (NF1)-deficient mice develop normal nerve. However, only after a sciatic nerve injury did loss of *Nf1* expression induce tumorigenesis, providing evidence that microenvironment signals at the wound site can be tumor-promoting [63]. The direct mechanism of transformation and the environmental signals involved with our observed tumorigenesis have yet to be elucidated, it is most likely that key molecules found in both myogenesis and neurogenesis are potential candidates. Loss of *Nf1* expression and mutations in the p53 tumor suppressor gene has been reported in many of the human and animal models of MPNSTs. However, we found no significant alteration in *Nf1* gene expression or any transformation mutations in the *Trp53* gene in our cells pre- and post-transplantation. Our results are in agreement with molecular analysis performed in a study by Strauss et al. [64], reporting the retention of *Nf1* gene expression in sporadically occurring human Triton tumors, suggesting that loss of *Nf1* expression is not required for the development of MPNSTs. Also, key observations in a mouse model of NF1 show that tumors only developed in a heterozygous (*Nf1* ^+/−^) background, implicating other *Nf1* ^+/−^ cell types in neurofibroma formation and *Nf1* loss of heterozygosity (LOH) in the Schwann cell lineage is necessary, but not sufficient, to elicit neurofibromas [65], [66]. Interestingly, our findings are also in accordance with an extensive study by Verdijk’s group that included a comprehensive review of literature, plus 145 MPNST cases over 36 years, assessing the *Trp53* gene mutation frequency. The results of their study indicate that in contrast to current animal models of MPNSTs, *Trp53* mutations are relatively rare in human MPNSTs. They also found no correlation between p53 immunostaining or *TP53* mutation with sex, age, NF1 status, or tumor location, suggesting a relatively minor role of this gene in MPNST tumorigenesis [67]. We believe our results are in accordance with the findings obtained from human patients, and consequently, our mouse model may be more relevant to human MTT at the cellular and molecular level. Therefore, studies are underway to gain a better understanding of signaling molecules and pathways involved in this rare and complex tumor.

Notably, the tumorigenesis observed here, though reproducible, was not a mutually exclusive event. The mice that developed Triton tumors simultaneously displayed complete sciatic nerve regeneration and regeneration within the skeletal muscle. Furthermore, this transformation could be abrogated when the cells were differentiated beyond the ability to respond to local signals or when their ability to receive the differentiation signal was impaired. In this case, differentiating the cells toward the neurogenic lineage prior to implantation abrogated tumorigenesis. We believe that the ability to halt transformation by removing the cells’ ability to respond to environmental cues (by differentiating our cells prior to implantation) is important. Not only does this finding substantiate the involvement of environmental signals in the transformation of MDSPCs, it also provides us with ideas as to how to avoid transformation. For example, by using a synthetic “nerve guide” [68], the MDSPCs may be protected from non-neuronal environmental signals and “forced” to only undergo neurogenic differentiation, while avoiding an oncogenic phenotype later in the regeneration process.

In summary, our results demonstrate key findings that murine muscle-derived stem/progenitor cells can functionally regenerate peripheral nerve; however, these cells can undergo spontaneous transformation at the site of nerve injury. Furthermore, this transformation occurs in an environment and time-dependent fashion and differentiating the cells prior to implantation eliminates transformation. Our results also provide evidence that experiments in tissue engineering and regenerative medicine should be evaluated over a prolonged period of time, as the transformation observed here occurs after 11 weeks post-implantation, several weeks after complete histological regeneration. We believe that in an effort to understand the origin of the perceived niche-specific oncogenesis, using this unique mouse model, we may reveal the biomarkers associated with human MTT and potentially identify novel therapeutic strategies.

## Supporting Information

Figure S1 (DOCX)
