# Supplementary material for: The Microenvironment-Specific Transformation of Adult Stem Cells Models Malignant Triton Tumors
Source: PLoS One. 2013 Dec 9;8(12):e82173. doi: 10.1371/journal.pone.0082173 (PMC3857244; doi:10.1371/journal.pone.0082173)

SUPPLEMENTARY INFORMATION

**The Microenvironment-Specific Transformation of Adult Stem Cells Models Malignant Triton Tumors**

Mitra Lavasani, Jonathan B. Pollett, Arvydas Usas, Seth D. Thompson, Aaron F. Pollett, Johnny Huard

**Figure S1.** To investigate the number of undifferentiated MDSPCs before the transformation was initiated, longitudinal sections of the hindlimb, including the regenerated nerve, were immunostained for *nLacZ* (red) and Sca-1 (green). At 5 weeks post-implantation, approximately 70% of *nLacZ*-positive donor cells present in the regenerating sciatic nerve were Sca1-positive (arrows), indicating that a large portion of donor MDSPCs remain as progenitor cells weeks after transplantation. Arrowheads indicate β-gal+/Sca-1- cells.


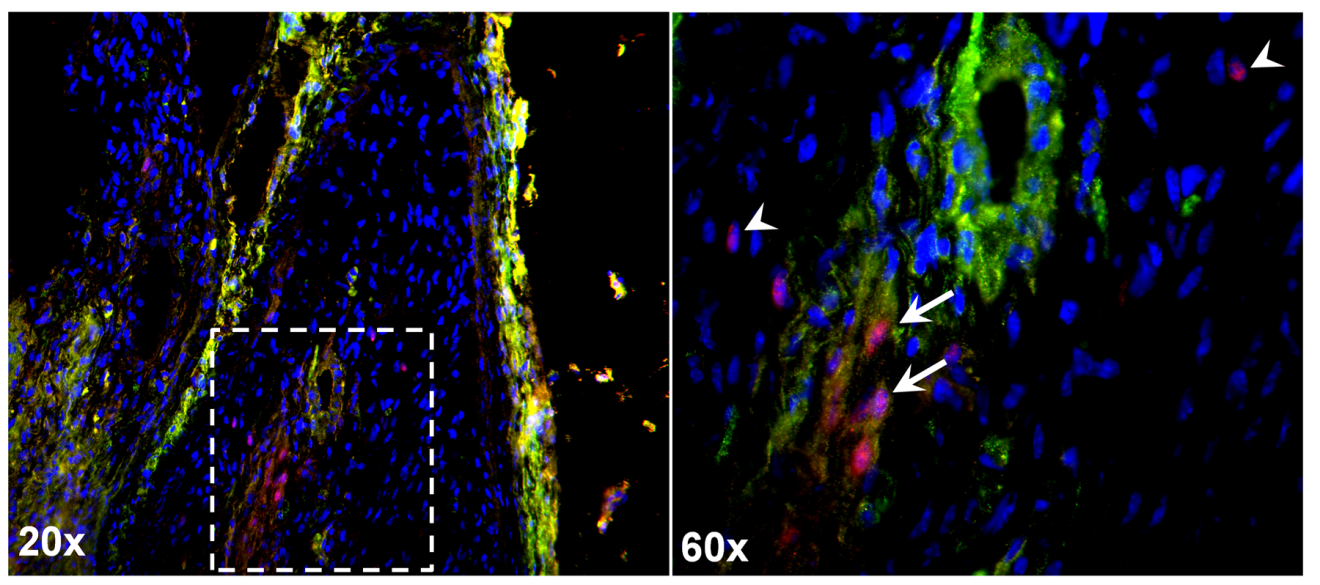

Supplement: Figure S1 — (DOCX) [file pone.0082173.s001.docx]
